# Congenital arteriovenous malformation of upper trunk in a child

**DOI:** 10.11604/pamj.2021.39.170.30143

**Published:** 2021-07-06

**Authors:** Rohan Kumar Singh, Prerna Anup Patwa

**Affiliations:** 1 Department of Radiodiagnosis, Jawaharlal Nehru Medical College, Datta Meghe Institute of Medical Sciences, Sawangi (Meghe), Wardha, India

**Keywords:** Arteriovenous malformation, trunk, congenital, pediatric

## Image in medicine

A 2-year-old male child was brought by his mother to outpatient Department of Surgery with chief complains of a swelling in the upper back on left side since birth. Further history suggested, the swelling was small in size since birth with associated mild tenderness and it gradually started increasing in size. On clinical examination, the swelling was pulsatile in nature, soft in consistency with mild reddish discolouration and ulceration of the overlying skin; however, there was no local rise of temperature. The swelling was superficial to the muscle plane and measured approximately 5.5 x 4.7 cm in size. For further investigation, the child was referred to the Department of Radiodiagnosis for ultrasonography. On ultrasound, there were multiple anechoic cystic areas showing increased vascularity on colour Doppler.

**Figure 1:**
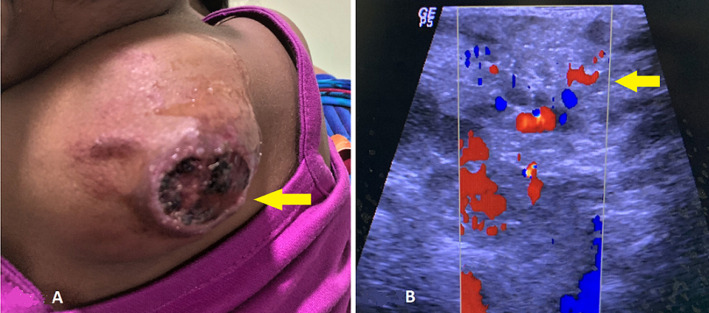
A) clinical image of the upper back of the child showing a large reddish mass lesion with ulceration (arrow); B) ultrasound image showing multiple anechoic cystic areas with Doppler showing increased vascularity (arrow) - arteriovenous malformation

